# Step-adaptive sound guidance enhances locomotor-respiratory coupling in novice female runners: A proof-of-concept study

**DOI:** 10.3389/fspor.2023.1112663

**Published:** 2023-03-01

**Authors:** Eric Harbour, Vincent van Rheden, Hermann Schwameder, Thomas Finkenzeller

**Affiliations:** ^1^ Department of Sport and Exercise Science, Paris Lodron University of Salzburg, Salzburg, Austria; ^2^ Department of Artificial Intelligence and Human Interfaces, Paris Lodron University of Salzburg, Salzburg, Austria

**Keywords:** breathing strategies, breathing techniques, locomotor-respiratory coupling, synchronization, running, entrainment

## Abstract

**Introduction:**

Many runners struggle to find a rhythm during running. This may be because 20–40% of runners experience unexplained, unpleasant breathlessness at exercise onset. Locomotor-respiratory coupling (LRC), a synchronization phenomenon in which the breath is precisely timed with the steps, may provide metabolic or perceptual benefits to address these limitations. It can also be consciously performed. Hence, we developed a custom smartphone application to provide real-time LRC guidance based on individual step rate.

**Methods:**

Sixteen novice-intermediate female runners completed two control runs outdoors and indoors at a self-selected speed with auditory step rate feedback. Then, the runs were replicated with individualized breath guidance at specific LRC ratios. Hexoskin smart shirts were worn and analyzed with custom algorithms to estimate continuous LRC frequency and phase coupling.

**Results:**

LRC guidance led to a large significant increase in frequency coupling outdoor from 26.3 ± 10.7 (control) to 69.9 ± 20.0 % (LRC) “attached”. There were similarly large differences in phase coupling between paired trials, and LRC adherence was stronger for the indoor treadmill runs versus outdoors. There was large inter-individual variability in running pace, preferred LRC ratio, and instruction adherence metrics.

**Discussion:**

Our approach demonstrates how personalized, step-adaptive sound guidance can be used to support this breathing strategy in novice runners. Subsequent investigations should evaluate the skill learning of LRC on a longer time basis to effectively clarify its risks and advantages.

## 1. Introduction

Breathing pattern, the temporal and mechanical characteristics of breathing, can reveal deep psychophysiological insights during running. For example, breathing rate (BR; respiratory frequency) is highly correlated with perceived effort (1) and dyspneic sensation (2) during exercise. Thus, recent publications (3, 4) have called for increased attention towards breathing as a key indicator in health and sport.

Locomotor-respiratory coupling (LRC), the synchronization of movement and breath, is a component of exercise breathing pattern that directly affects BR. LRC occurs when movement entrains breathing; it is a synchronization phenomenon of frequency and/or phase (5). In running, this implies a whole-integer ratio between BR and step rate (SR) and/or step to breath cycle (i.e., stepping precisely on expiration). LRC may be performed consciously or unconsciously and is more prevalent in experienced runners (6, 7). The exact mechanisms are still debated; it likely results from an interplay of mechanical and neurophysiological constraints (6). LRC during exercise is perturbed by a number of factors: it is more prevalent during higher intensities, during activities with greater postural muscle activity, higher rhythmicity, and with external stimuli (e.g., music) (6, 7).

Studies have reported that performing LRC decreases oxygen consumption, increases movement economy, and reduces dyspnea (8–12). Other studies found no such benefits (13–15). This could be due to the mix of activities investigated - running is notable because of its large impact forces and the postural demands of the trunk muscles. Indeed, Daley, Bramble, and Carrier (16) studied treadmill running and found that impact forces likely contribute substantially to ventilatory flow (up to 10%–12%) when footstrike and breath onset are precisely synced (“ in phase”). They attributed this to the “visceral piston”; downward momentum of the abdominal viscera directly pulling on the diaphragm can be additive to ventilation. Unfortunately, there is a paucity of research on LRC and its metabolic and perceptual implications in running. This is additionally complicated by the potentially harmful effects of spirometry masks on breathing pattern (17, 18). Thus, there is a need to bridge the gap by providing tools that enable the study of LRC in field running.

Additionally, frequency coupling alone may have beneficial effects especially relevant for the novice runner. Since SR is generally quite stable during running (19) and lower BR variability (BRV) is associated with higher exercise performance (20), LRC may support novices in regulating BR and exercise intensity by aiding in self-awareness (21). Finally, LRC at odd ratios (e.g., 5:1 steps per breath) could contribute to a reduced risk of side stitch. Up to 70% of runners experience this unpleasant sensation each year (22), perhaps contributing to pain or exercise cessation. Since repeated expiration on ipsilateral strides may trigger phrenic nerve irritation, this could be avoided by deliberate coupling of breath of contralateral steps (21).

### 1.1. Guiding LRC

There is scant information available regarding how to guide runners to perform LRC. Several studies have used custom biofeedback applications with appreciable results (11, 23); other studies instructed participants to count steps per breath (24, 25). In a notable book on this subject, Coates and Kowalchik (26) advocate a multi-step approach with verbal coaching, static and dynamic exercises, and specific LRC ratio recommendations.

Sound instruction has been frequently and successfully used in laboratory studies to induce LRC quickly and consistently. Some studies found that fixed-tempo audio stimuli increased LRC compared to control (silent) conditions (9, 27). This was explained by the “anchoring effect”: external stimuli can entrain physical processes such as bimanual coordination and, notably, breathing (28). Another study used in-shoe pressure sensors during a cycling task to create individualized sound cues and instructed participants to consciously couple their breathing to their cadence (11); they found an increase in LRC occurrence from about 55% to 70% of total run time.

Unfortunately, these highly-standardized, equipment-intensive laboratory setups are likely not suitable to guide LRC in the field nor commercially available as biofeedback applications. Moreover, sound tempo should be mindfully selected; fixed metronome sounds could be harmful to use during exercise to entrain movement or breathing. While constant, isochronal sounds may appear to be a predictable, effective stimulus for entrainment, they oppose the natural complex and fractal behavior of the stride and breathing rhythms in healthy humans (29, 30). Since deviations from an individual's preferred step rate (SR) could be metabolically disadvantageous (31), or even increase injury risk (32), it might be preferable to continuously adapt the sound instruction to the runner's real-time SR (33).

Real-time step frequency feedback during running has distinct effects on stride biomechanics and psychological states. A recent review extensively detailed the broad set of mechanisms responsible for human's sound-movement entrainment and found decreased negative affect, increased time to exhaustion, and decreased stride time variability when synchronizing to an external rhythmic stimulus (34). Hence, introducing step-adaptive sounds might itself affect the runner. Adding breathing cues could increase the cognitive load associated with such guidance by introducing a dual-task problem, which is known to perturb normal gait dynamics (35). As step sound cues are known to increase the likelihood of LRC, it is preferable to compare step-only feedback to LRC sound guidance to effectively separate the effects of LRC guidance from that of step-adaptive audio.

While LRC could be helpful to all runners, females are especially predisposed to realize larger benefit since they are more likely to experience respiratory limitations compared to males (36). This has been attributed to morphological (i.e., relatively smaller airways) and functional (i.e., higher metabolic work of breathing at equal ventilation) differences (37). Furthermore, novice runners may see additional benefit, since they can experience high levels of exertion and risk for exercise-induced breathlessness at relatively low exercise intensities (38, 39).

### 1.2. Aims & hypotheses

The primary aim of this study was to examine the effect of step-adaptive LRC sound guidance vs. step-only feedback in the field and laboratory upon frequency and phase coupling in novice female runners. We hypothesized that such guidance would induce increases in frequency and phase coupling and decreases in breathing pattern variability. Second, we aimed to investigate individual parameters such as instructed LRC ratio (e.g., odd vs. even ratios) and deviation from baseline BR. We hypothesized that instruction adherence would be lower with odd ratios and with larger deviations from the individual's baseline BR as measured during the control visits.

## 2. Methods

### 2.1. Sample

A sample of 17 female beginner to moderately-experienced runners volunteered to participate in this study (Table 1). These volunteers were chosen as a varied representation of the target group (see below). One participant was excluded due to substantial LRC in the T1 visit.

**Table 1:** Participant characteristics and description.

Subject	Age (y)	Training Frequency	Mean run duration (min)	Mean run distance (km)	Self-selected pace (km/h)
1	34	1–2/month	30	5	10.0
2	28	1–2/month	35	6	9.2
3	27	1–2/week	45	8	10.2
4	29	1–2/month	60	10	11.8
5	30	1–2/month	30	5	8.7
6	33	1–2/month	50	7	10.4
7	31	1–2/week	40	6	10.2
8	25	1–2/month	40	7	10.9
9	35	<1/month	20	3	9.7
10	32	1–2/month	35	5	8.7
11	32	<1/month	10	1	7.0
12	31	1–2/month	35	5	10.0
13	29	<1/month	35	6	8.8
14	27	1–2/week	30	5	10.8
15	33	<1/month	25	3	8.4
16	28	<1/month	35	5	9.2

Training frequency, mean run duration, and mean run distance information derived from self-reported questionnaires. Self-selected pace measured as mean pace throughout the second half of T1 outdoor run. One participant was excluded due to observed coupling during the experiment; see results.

Female runners were chosen not only because of the possible benefits proposed above, but also because this project was part of a larger research initiative seeking to do research together with females as they are understudied in sports research (40). None of the participants indicated to use a special breathing technique while running; however, three participants indicated that they breathe deeper when feeling “side stitches”.

Ethical approval was granted by the Ethics Committee of University of Salzburg (reference number: GZ 13/2021) and participants gave their informed consent. Participants filled in a pre-questionnaire to including demographic information, as well as running, sport, and breathing experience. The final sample was selected based on the following criteria:

• participants are not familiar with nor actively perform paced breathing techniques (including LRC) during running
• participants self-identify as beginner to intermediate runners
• participants can run for at least 30 min without stopping

None of the participants indicated any experience or familiarization with LRC during running.

### 2.2. Breathtool app

Breathtool is a custom-designed, Android-based mobile application designed to assist runners with LRC. Through simple audio the runner is instructed to either breathe in or out at every *n*th step. As described above, since SR-adaptive audio is preferable to instruct LRC, Breathtool adapts the audio instruction based on instantaneous SR. The stock Google/Android step detection was utilized and smoothed over a 5-step moving average to which the sounds were synchronized and generated. A short, high-pitched tone was played on steps indicating inhalation, while expiration was indicated by a low-pitched tone. Pilot testing showed the application to be sufficiently accurate against a reference tibia-mounted accelerometer with limits of agreement centered around a SR difference of 0 [95% confidence interval = −3.33, 2.97] and a trivial bias = −0.18 [−0.416, 0.056] steps per minute.

From a simple user interface, basic LRC parameters (including steps per inhale: exhale) can be chosen and audio is streamed *via* Bluetooth to headphones or a speaker. The app also logs GPS data locally. The same phone (Samsung Galaxy S8+, Android 9) was used for all conditions, and placed securely in a waist belt on the caudal side of the pelvis for all participants.

### 2.3. Instruments

**Figure 1:**
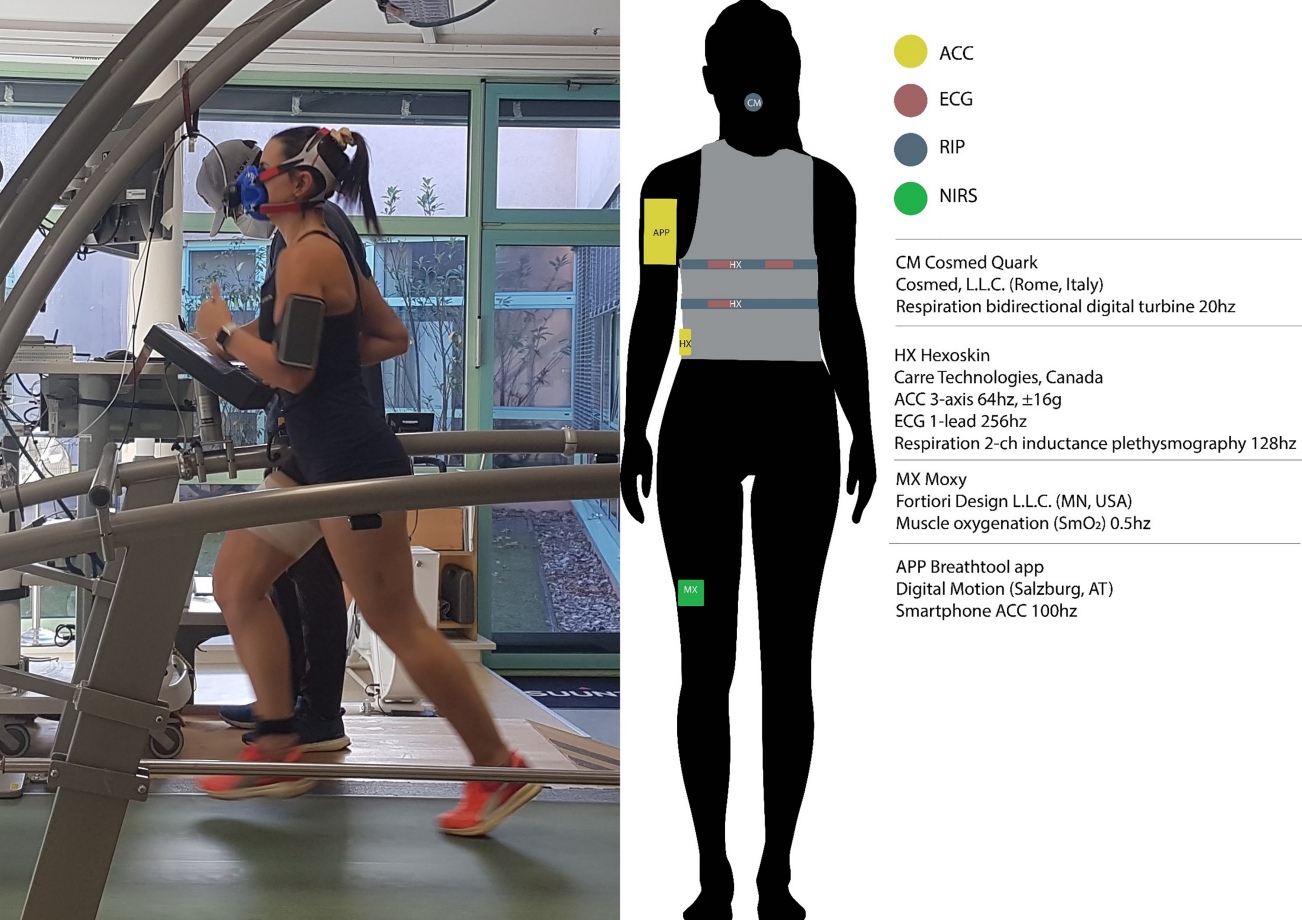
Experimental sensor setup with Hexoskin smart shirt, Breathtool app, and Cosmed spirometer. Note that this upper arm placement of the phone was not used for the actual experiment (instead, waist). Permission was obtained for use by the persons in the photo.

Participants wore a Hexoskin smart shirt (HX; Carré Technologies, CAN) in all conditions to gather respiration (dual thoracic and abdominal stretch sensors, 2 channel respiratory inductance plethysmography, 16-bit, 128 Hz), heart rate (1 Ch ECG, 12bit) and accelerometry (3-axis, 64 Hz, range ±16 g) data (Figure 1).

During the indoor conditions, participants ran on a treadmill ergometer (h/p cosmos sports, Traunstein, Germany) calibrated to manufacturer's specifications. The Moxy (Fortiori design, LLC, MN, USA) near-infrared spectroscopy sensor was worn on the right vastus lateralis. These data are omitted from this report.

Questionnaires related to rating of perceived exertion, dyspnea, subjective vitality, user experience, and prior music experience were used to gather subjective data at various timepoints. RPE data is reported in Supplementary Tables. As the other data are not the focus of this article, they are omitted from this report.

### 2.4. Study design

**Figure 2:**
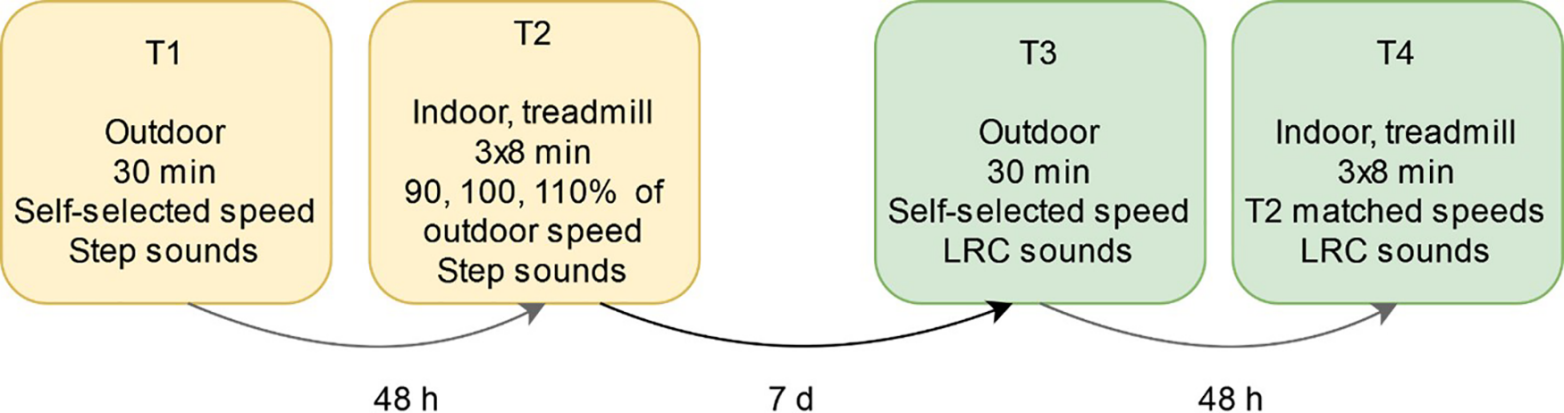
Experimental protocol for T1–T4.

A sequential within-subjects design was used to compare step-only and LRC guidance (Figure 2). This experimental design was developed with stepwise instruction and individual calibration to maximize instruction adherence and to minimize deviation from participant's natural BR. The first outdoor (T1) and indoor (T2) visits were intended to familiarize the runners to the Breathtool app with single-tone step sounds and to measure each runner's baseline individual breathing pattern. We speculated that step-only sounds would have negligible effects on LRC occurrence since this cohort was inexperienced both in running and LRC. As we aimed to contrast spontaneous LRC occurrence with step sounds vs. LRC guidance in the experimental visits, participants were excluded if they showed deliberate LRC performance in T1.

Upon arrival, participants were fitted with a HX shirt and then performed a silent resting phase for 5 min to capture resting BP and heart rate data. This standardized measurement was performed before and after all study visits also to encourage a similar psychophysiological state between runs.

#### 2.4.1. Step-only feedback

In T1 participants ran continuously for 30 min outdoors on a 1 km forest path. During a one-lap warmup, one researcher accompanied the runner with a Bluetooth speaker to explain the application and sound instructions. Participants wore Bluetooth headphones for the remainder of the run. There was no explicit breathing instruction; runners were only told to “step to the beat”. The runners were instructed to run continuously at a steady pace that they thought they could sustain for 30 min.

In the second visit approximately 48 h later, participants ran on a treadmill at 90%, 100%, and 110% (8 min each) of the speed derived from the mean pace of the T1 run. These speeds were used to examine LRC at preferred and non-preferred speeds. Runners completed a warmup including 3 min walking at 4 km/h and 5 min jogging at 7.5 km/h, during which they were refamiliarized to the step-only feedback. They had 3 min rest after warmup and between each experimental condition.

#### 2.4.2. Breathtool LRC guidance

About one week after the T1 and T2 visits, participants returned for identical running sessions with added LRC instruction. The mean BR and SR of the final 50% of the T1 and T2 runs were used to calculate the instructed LRC parameters for T3 and T4, respectively. Several other considerations were made for LRC instruction:

1. LRC ratio (steps per breath) was calculated as the quotient of SR/BR rounded to the nearest integer (e.g., for a SR/BR of 170/25 = 6.8 would be rounded up to 7 steps per breath).
2. Inhale: exhale ratio was preferentially kept equal. If odd number of steps per breath was estimated, then always 1 step per exhale more was chosen (e.g., for 7 steps per breath, a ratio of 3:4 steps per inhale: exhale)
3. For the T4 visit, LRC ratios either decreased 1 step/breath or remained unchanged between speed conditions (e.g., when 3:4 was instructed, the next run would be 3:4 or 3:3, but never 4:4 (increasing steps/breath) or 2:3 (decreasing the ratio by 2 steps/breath or more).
4. The instructed LRC ratios were never instructed faster than 2:2.

Participants were introduced to Breathtool LRC guidance through a detailed researcher-led familiarization. LRC sounds were played over the Bluetooth speaker as the participants were asked to step in place and breath along with the instructed rhythm. This was repeated during running. The researcher cued the correct LRC by (1) counting steps by breath phase (e.g., *in*-2-3-*out-*2-3 for 3:3) and (2) breathing aloud to the app sounds. The participant was encouraged to run independently in the experiment once the researcher observed clear understanding, which was observed after the warmup for all participants.

In T3, participants ran with one LRC ratio during the entire 30 min outdoor run. The procedure and instructions were identical to T1, except for the addition of LRC instruction and suggestion to attempt to replicate the RPE and pace of the T1 run (Supplementary Table S1). In T4, participants ran on the treadmill at identical speeds to T2 but with LRC ratios derived from each speed condition in T2. This resulted in diverse LRC ratios (i.e., slow, fast, even, odd) between participants.

The principal investigators utilized standardized verbal instruction during the LRC familiarization. Similar to Coates and Kowalchik (26), participants practiced LRC while stationary (foot tapping) and walking until they demonstrated conceptual understanding. Next, while running, the researchers counted breath and steps; for example, a 3:3 ratio was voiced, “in-2-3-out-2-3”. Also, the researcher performed exaggerated breathing sounds in-phase with the runner's steps to emphasize the synchronization. The running familiarization lasted exactly 1 km for all participants; both researcher and runner acknowledged subjective understanding prior to all runs.

### 2.5. Data processing

HX raw data were trimmed to reflect representative areas of interest for overall instruction adherence estimation (see section “Instruction Adherence”); for T1/T3 outdoor conditions, the first and last 30 s were trimmed, and the rest of the run separated into equal quartiles. T2/T4 indoor data were trimmed from 30 s after start (exactly at sound start) to the last minute (e.g., minutes 0.5–7).

#### 2.5.1. Event detection

HX respiration data was processed using a custom-built algorithm (41) in MATLAB (MATLAB Version 2021a, MathWorks, Inc., Natick, United States). Step detection from HX accelerometer data was performed using an adapted version of the algorithm by Benson et. al (42).. Previous investigations revealed these methods to be extremely accurate (<5% bias) during running (41, 43).

#### 2.5.2. Locomotor-respiratory coupling

Previous investigations investigating LRC have used diverse methods for calculation and comparison, so we followed the best practice recommendations of Stickford A.S. and Stickford J.L. (6) by choosing techniques to quantify both types of coupling (frequency and phase).

#### 2.5.3. Frequency coupling and instruction adherence

LRC ratio was calculated on a per-breath basis using the quotient of average SR (over one breath cycle) and BR (five-breath rolling average). Frequency coupling was quantified similar to a previously-reported adherence estimation (43, 44). Attachments and detachments were defined *a priori* as five or more consecutive breaths inside or outside, respectively, of 5% of the instructed LRC ratio. For example, for an instructed LRC ratio of five, an attachment was flagged if five consecutive breaths had a ratio between 4.75–5.25 steps per breath. These were calculated from exact sound start for all conditions. We chose to report the percentage of run time “attached” or “detached” (vs. total run time). Note that runners can be neither attached nor detached, so they do not necessarily sum to 100%. The same routine was used to quantify spontaneous LRC occurrence to any whole-integer ratio in the step-only conditions.

#### 2.5.4. Phase coupling and entrainment

Phase coupling between each flow reversal (FR; onset of inspiration or expiration) and the nearest step was calculated using discrete relative phase and sine-circle maps as done previously (7). Specifically, relative phase was mapped from 0 ± 180° with 0 representing foot strike. Then, resultant vectors were calculated for expiration and inspiration entrainment separately. Resultant vectors have a length (*ρ*) ranging from zero to one (“stability”), with one indicating perfect entrainment. The resultant vector angle (*θ*) represents the average phase angle (“timing”), where a negative angle (−180–0°) indicates breaths before footstrike and positive (0–180°), after footstrike. The MATLAB toolbox circular statistics (45) was used to evaluate entrainment stability and timing for expirations and inspirations.

#### 2.5.5. Breathing pattern variability

BR variability (BRV) was quantified using the coefficient of variation (CV) of BR (46). Additionally in the breathing time series, “reset breaths” were flagged if a single breath was more than twice as deep or less than half of the BR of the previous one minute average. This measure was inspired by the psychophysiological construct of sighing and respiratory variability (47). The reset breath rate (reset breaths/min) was calculated as a quantitative measure reflecting respiratory discomfort and instability.

### 2.6. Statistical analysis

A within-subjects ANOVA with repeated measures was performed in MATLAB to assess differences in outcome variables LRC frequency and phase coupling with factors: trial (T1 vs. T3) and run quartile. The same analysis was performed for T2 vs. T4 with factors trial and speed condition (90%, 100%, 110%). All variables met the *a priori* requirements for normality, assessed *via* Q-Q plots and Kolmogorov–Smirnov tests, and homogeneity of variances, assessed *via* Mauchly's test of sphericity. For the phase coupling resultant vectors, the Hodges–Ajne test was used to confirm nonuniform vector direction to indicate significant entrainment. Post-hoc Bonferroni-corrected pairwise comparisons were used to determine where significant differences existed in run quartile (T1 vs. T3) and speed condition (T2 vs. T4) between trials. We also assessed each possible two-way interaction in all comparisons.

A deeper analysis of T3 and T4 instruction adherence was performed using an almost identical ANOVA approach to that outlined above. ANOVA with repeated measures was used to assess whether outcome variables frequency and phase coupling were affected by factors 1. instructed LRC ratio, 2. even vs. odd ratio instruction, and 3. absolute deviation from T1/T2 BR. Finally, discrete relative phase was calculated and separated for periods during attachment (>10 s) and outside of attachment in order to estimate the effect of frequency coupling onto phase coupling within individuals.

Data are presented as mean ± standard deviation (95%) unless otherwise stated. Partial eta squared effect sizes were calculated using the formulas provided by Lakens (48) and interpreted as small ($\eta_{p}^{2\;}\;=\;0.01$), medium (0.06), and large (>0.14) (49).

## 3. Results

### 3.1. Outdoor runs: T1 step sounds vs. T3 LRC sounds

There was no significant change in running pace between the T1 and T3 outdoor runs [F(1,15) = 0.68, $\eta_{p}^{2\;}\;<\;0.01$, *p* = 0.80]. The average BR measured in T1 was 38.1 ± 7.4, resulting in approximate LRC ratios of 4.6 ± 0.8 steps per breath. Consequentially, the prescribed LRC ratios for T3 were either four (*n* = 5), five (*n* = 8), or six (*n* = 3) steps per breath (Table 2).

**Table 2:** Group summary of primary locomotor-respiratory coupling variables between outdoor conditions.

	Pace (km/h)	Ratio (SR/BR)	BR (bpm)	% time attached	% time detached	BRV (%)	Exp *ρ* (0–1)	Exp *θ* (rad)	Insp ρ	Insp θ	RB/min	SRV (CV; %)
T1	9.6 ± 1.2	4.6 ± 0.8	38.1 ± 7.4	26.2 ± 10.7	16.5 ± 3.6	19.7 ± 3.4	0.20 ± 0.12	−0.45 ± 1.3	0.23 ± 0.12	−2.27 ± 0.81	0.6 ± 0.6	3.5 ± 1.9
T3	9.5 ± 1.0	4.9 ± 0.7*	35.2 ± 5.3	69.9 ± 20.0*	8.3 ± 4.1**	14.7 ± 4.9*	0.43 ± 0.18*	3.03 ± 0.84*	0.39 ± 0.19	2.61 ± 0.92	0.4 ± 0.3	4.3 ± 3.6

T1, outdoor, step sounds; T3, outdoor, coupling sounds; SR, step rate; BR, breathing rate; bpm, breaths per minute; BRV, breathing rate variability; Exp, expiration; Insp, inspiration; *ρ* = entrainment stability; θ = entrainment timing; RB, reset breath; SRV, step rate variability; CV, coefficient of variation.

* *p* < 0.05.

** *p* < 0.001 compared with step sound visit.

#### 3.1.1. Frequency coupling and variability

**Figure 3:**
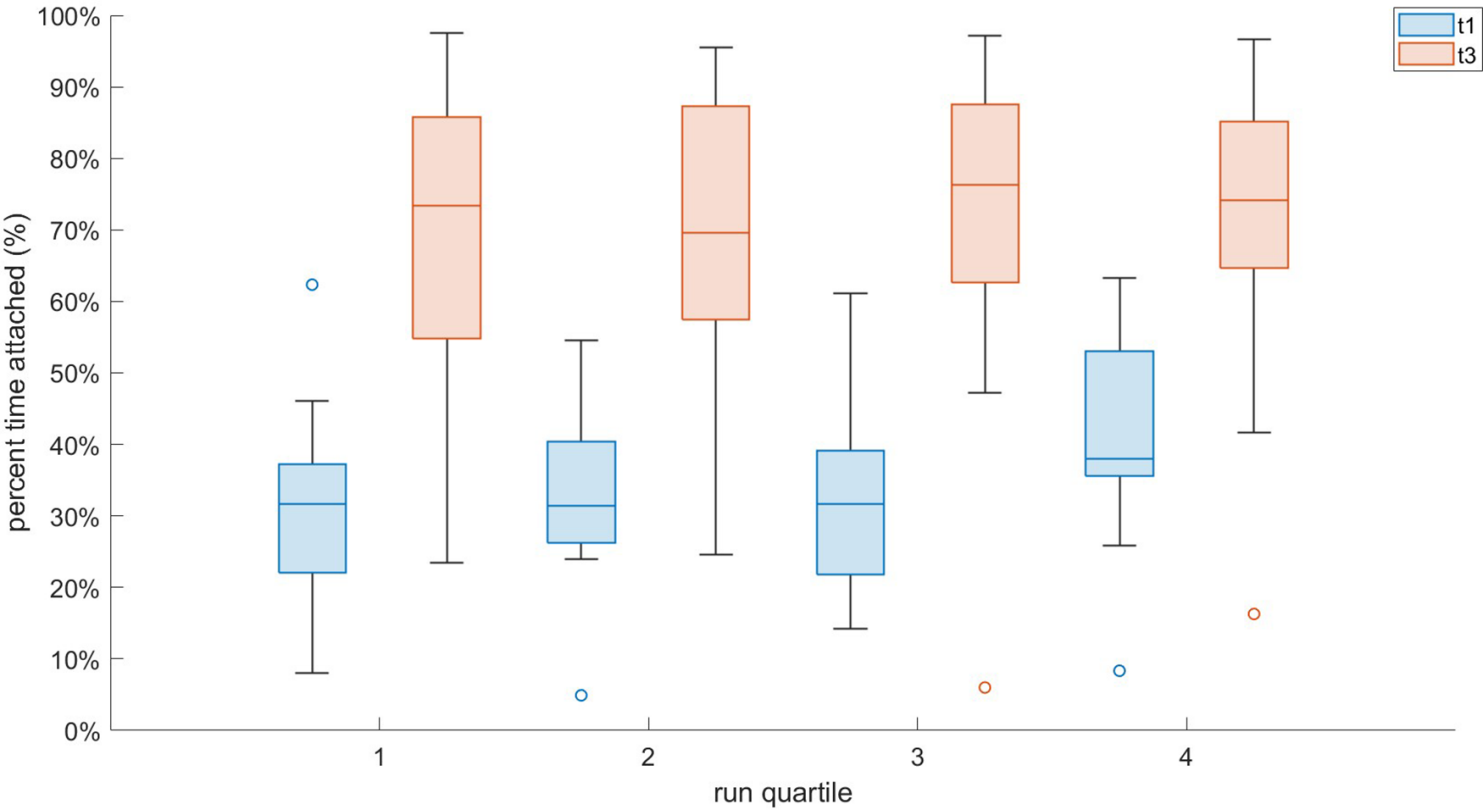
Interval plot of T1 vs. T3 attachment for all participants across equal quartiles of the run.

**Figure 4:**
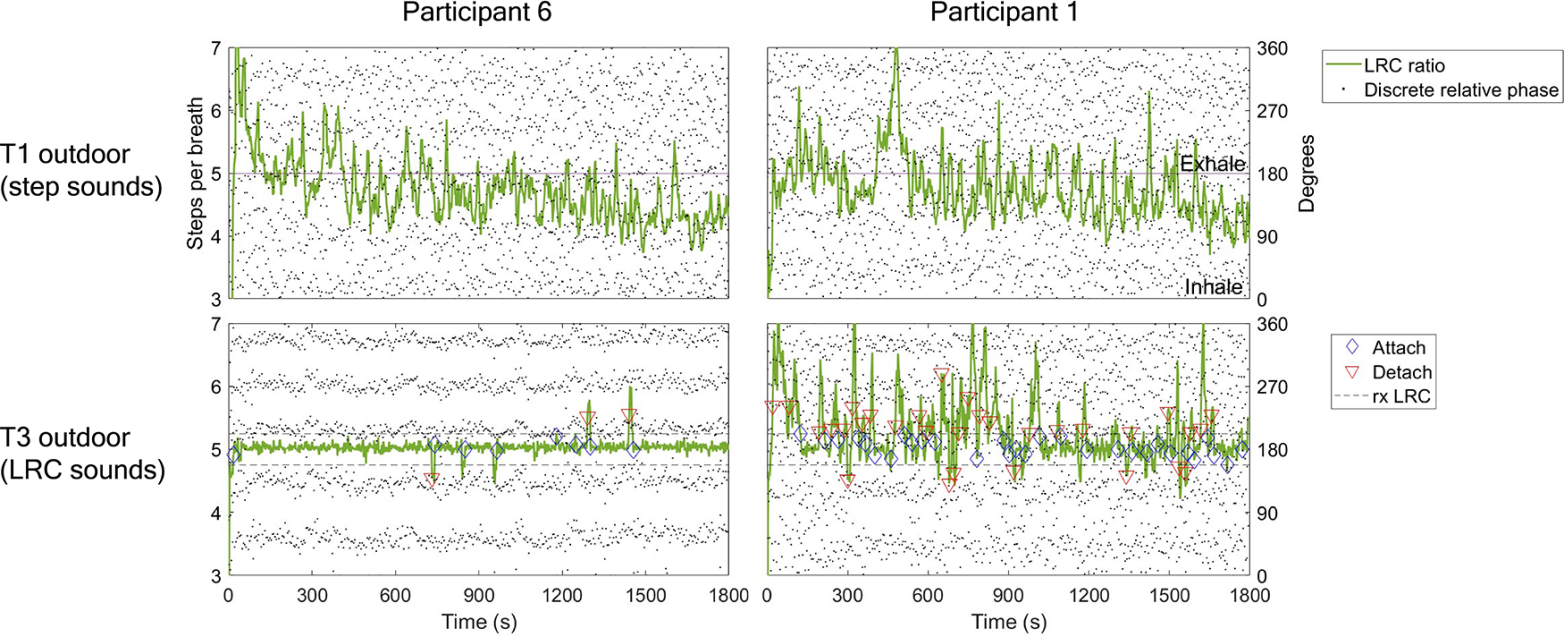
Example LRC ratio, phase synchrogram, and attachment plot for two participants during T1 and T3 outdoor runs. LRC ratio quantified using SR/BR quotient (steps per breath). Phase synchrogram shows relative phase of each step within the breath cycle (0–360 degrees, 0 denotes inhalation, 180, exhalation). Steps nearest inhalation are calculated relative to inhalation (270–360, 0–90), and same with steps closest to exhalation (90–270 degrees). This is done for visual assessment only. Note that this differs from the sine circle map and circular statistics approach, which uses relative phase of breath to step for statistical efficacy. Attachment calculation described in methods section as five or more breath cycles within 5% of prescribed LRC ratio. First column shows participant 6 with T1 % time attached = 9.4%; T3, 87.7%. In the top panel note the highly variable LRC ratio throughout the run, starting between 6-7 steps/breath and then speeding up to around 5 steps per breath as BR increased. In the bottom panel note the strong adherence to the instructed ratio of 5 throughout most of the run. Parallel dot groups indicate consistent relative phase of steps within the breath cycle. Second column shows participant 1 with lower comparative LRC strength in T3 (LRC sounds). T1 % time attached = 24.2 %; T3, 51.8%. Note the large amount of attachments & detachments as well as inconsistent relative phase of steps within breath (random dots dispersion).

Frequency coupling was much stronger in T3 vs. T1, as participants were “attached” 69.9 ± 20.0 vs. 26.3% ± 10.7% of the run [F(1,15) = 15.86, $\eta_{p}^{2\;}\;=\;0.51$, *p* = 0.001; Figure 3] Figure 4 demonstrates a representative example comparison for two participants. Pairwise comparison revealed that each quartile of T3 was significantly different than the corresponding T1 quartile (*p* < 0.001). BRV also had a large difference [19.8 ± 3.4 vs. 14.7% ± 4.9% for T1 vs. T3, respectively; F(1,15) = 15.25, $\eta_{p}^{2\;}\;=\;0.50$, *p* = 0.002] and each pairwise comparison was significant (*p* < 0.001). There was no apparent effect of BR deviation magnitude from T1 upon T3 adherence.

#### 3.1.2. Phase coupling

**Figure 5:**
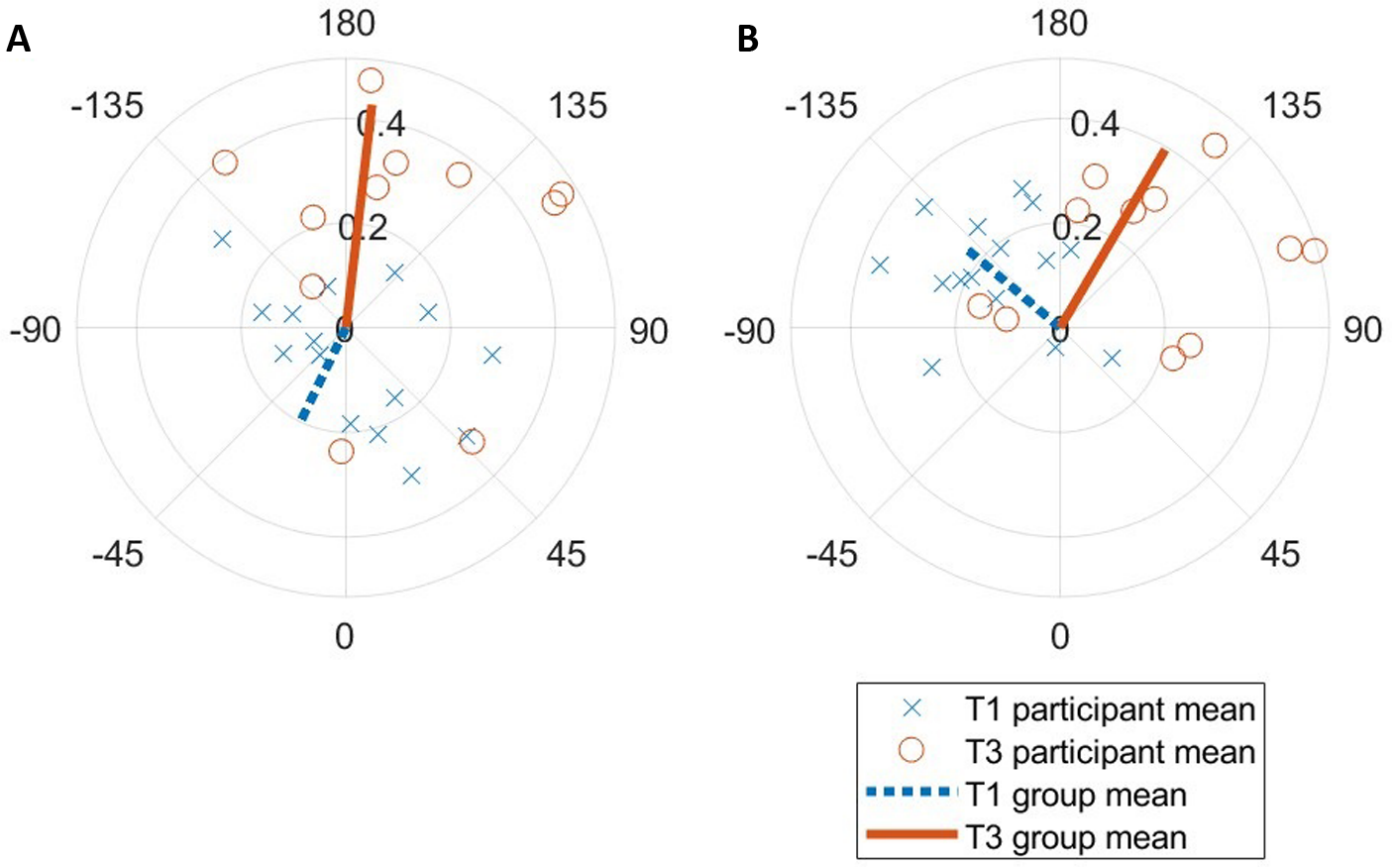
Sine-circle map of T1 vs. T3 (**A**) expiration and (**B**) inspiration entrainment resultant vectors relative to step cycle. Each marker represents the ensemble average for one participant over the entire run.

Phase coupling was much more stable in T3 vs. T1 for expirations [*ρ* = 0.43 ± 0.18 vs. 0.20 ± 0.12; F(1,15) = 7.60, $\eta_{p}^{2\;}\;=\;0.34$, *p* = 0.015; Figure 5], and all pairwise comparisons were significant (*p* < 0.001). A different effect was seen for inspirations; while there was a small nonsignificant difference between conditions [*ρ* = 0.39 ± 0.19 vs. 0.23 ± 0.12; F(1,15) = 0.45, $\eta_{p}^{2\;}\;=\;0.03$, *p* = 0.51], each pairwise comparison was significant (*p* < 0.02). There was a large difference in expiration timing *θ* [F(1,15) = 3.22, $\eta_{p}^{2\;}\;=\;0.13$, *p* = 0.023] across conditions, but not inspiration timing.

#### 3.1.3. Within-trial T3 LRC guidance analysis

**Figure 6:**
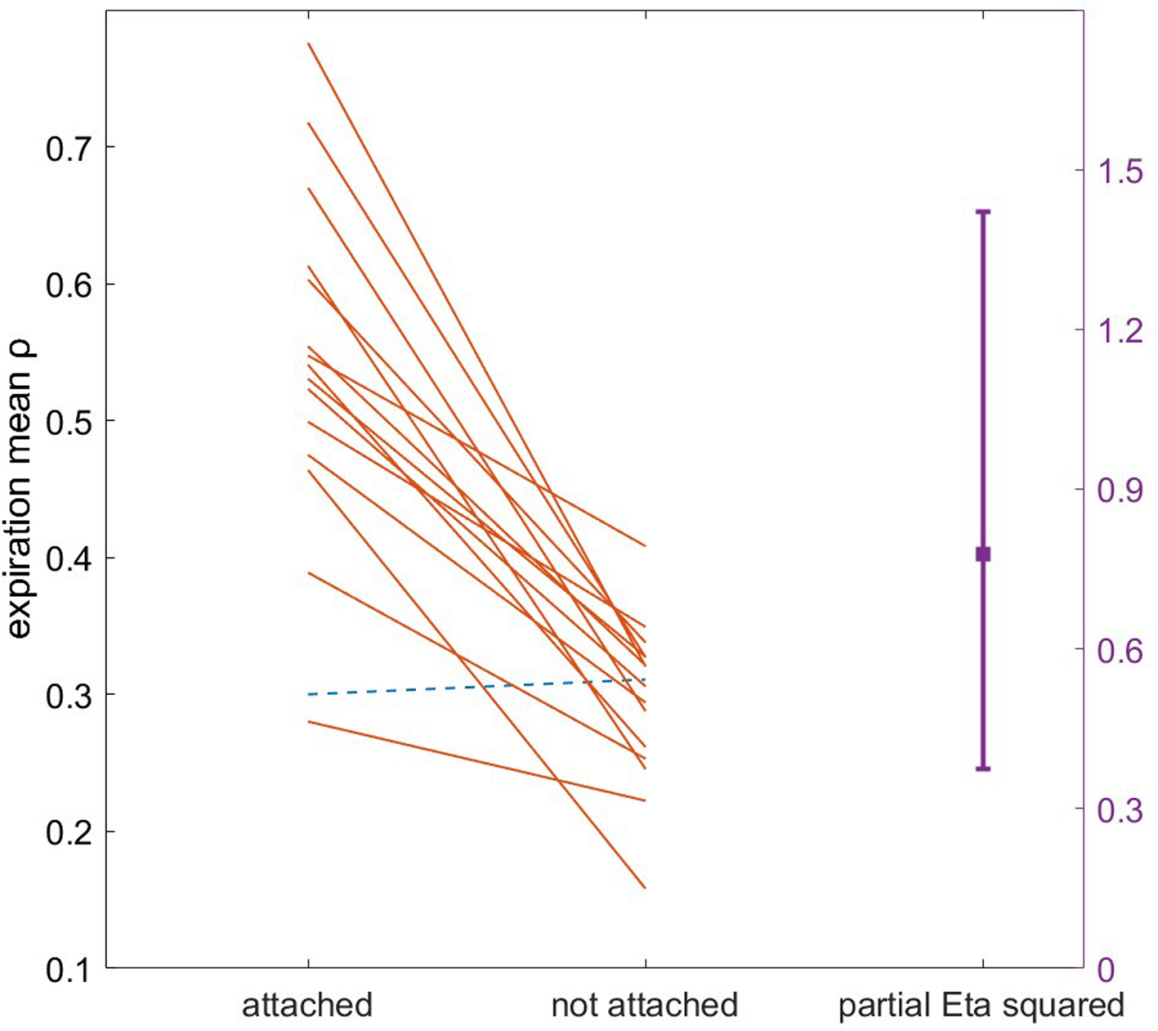
Gardner-Altman plot of T3 expiration entrainment stability *ρ* during attachment vs. unattachment. Each line corresponds to one participant. Note the single participant with small negative difference in entrainment when attached (dotted line).

No significant within-condition differences in attachment or detachment were detected between T3 quartiles. There was a large but nonsignificant effect of instructed ratio on attachment [F(2,15) = 3.51, $\eta_{p}^{2\;}\;=\;0.32$, *p* = 0.039], with pairwise comparisons trending towards better attachment to 2:3 ratio (*n* = 8) than 2:2 (*n* = 5; *p* = 0.037). Additionally, there were significantly more reset breaths in the first quartile vs. all others (*p* < 0.001). No apparent relationship was observed between odd vs. even ratios and other coupling adherence variables. Participants exhibited substantially higher entrainment stability *ρ* during attachment than when not attached (expiration, F(1,15) = 54.48, $\eta_{p}^{2\;}\;=\;0.78$, *p* < 0.001; inspiration, F(1,15) = 42.56, $\eta_{p}^{2\;}\;=\;0.74$, *p* < 0.001; Figure 6).

### 3.2. Indoor treadmill runs: T2 step sounds vs. T4 LRC sounds

In T2, the average detected ratios were 4.8 ± 1.5, 4.7 ± 1.4, and 4.3 ± 1.0 steps per breath for the slow, neutral, and fast speeds, respectively. In T4, the individually instructed ratios ranged from 4 to 8 steps per breath (Table 3).

**Table 3:** Group summary of primary locomotor-respiratory coupling variables between indoor conditions.

	Condition	Pace (km/h)	Ratio (SR/BR)	BR (bpm)	% time attached	% time detached	BRV (%)	Exp ρ (0–1)	Exp θ (rad)	Insp ρ	Insp θ	RB/min	SRV (CV; %)
T2	90%	8.1 ± 1.1	4.8 ± 1.5	36.5 ± 12.9	40.5 ± 24.8	13.6 ± 5.0	25.8 ± 31.7	0.32 ± 0.18	−2.78 ± 1.32	0.33 ± 0.24	−2.34 ± 1.05	0.8 ± 1.3	4.8 ± 1.1
	100%	9.0 ± 1.2	4.7 ± 1.4	37.9 ± 12.4	34.9 ± 23.7	16.6 ± 5.9	21.1 ± 14.3	0.28 ± 0.14	−2.45 ± 1.23	0.29 ± 0.22	−1.87 ± 0.75	1.3 ± 2.7	4.8 ± 1.3
	110%	9.9 ± 1.3	4.3 ± 1.0	41.0 ± 12.6	38.4 ± 22.6	14.8 ± 5.3	17.4 ± 5.6	0.29 ± 0.14	−1.32 ± 1.31	0.37 ± 0.20	−2.04 ± 0.62	0.8 ± 0.9	5.9 ± 2.4
	Mean	–	4.6 ± 1.3	38.5 ± 12.6	37.9 ± 23.7	15.0 ± 5.4	21.4 ± 17.2	0.30 ± 0.15	−2.18 ± 1.29	0.33 ± 0.22	−2.08 ± 0.81	1.0 ± 1.7	5.2 ± 1.6
T4	90%	8.1 ± 1.1	5.5 ± 1.2*	30.0 ± 6.2*	92.9 ± 6.8**	1.8 ± 2.2**	7.8 ± 3.0,	0.49 ± 0.25*	−2.45 ± 1.13	0.55 ± 0.16*	3.04 ± 0.83	0.3 ± 0.4	4.7 ± 0.9
	100%	9.0 ± 1.2	5.2 ± 1.0*	31.8 ± 5.8*	93.9 ± 8.0**	1.4 ± 2.5**	7.9 ± 4.2*	0.57 ± 0.21*	−2.23 ± 1.07	0.55 ± 0.18*	3.07 ± 1.14	0.2 ± 0.3	5.5 ± 2.6
	110%	9.9 ± 1.3	4.6 ± 0.6	36.2 ± 4.8	90.1 ± 10.3**	2.5 ± 2.5**	8.3 ± 2.6	0.52 ± 0.23*	−2.41 ± 0.85	0.50 ± 0.25	−2.86 ± 1.18	0.1 ± 0.2*	5.0 ± 1.4
	Mean	–	5.1 ± 1.0	32.7 ± 5.6	92.3 ± 8.4*	1.9 ± 2.4*	8.0 ± 3.3	0.53 ± 0.23	−2.36 ± 1.01	0.53 ± 0.20	1.08 ± 1.05	0.2 ± 0.3	5.1 ± 1.6

T3, outdoor, coupling sounds; T2, indoor, step sounds; T4, indoor, coupling sounds; SR, step rate; BR, breathing rate; bpm, breaths per minute; BRV, breathing rate variability; Exp, expiration; Insp, inspiration; *ρ* = entrainment stability; *θ* = entrainment timing; RB, reset breath; SRV, step rate variability; CV, coefficient of variation.

* *p* < 0.05.

** *p* < 0.001 compared with step sound visit.

#### 3.2.1. Frequency coupling and variability

Quantitative differences between step and LRC sound conditions were overall more pronounced indoors compared to outdoors. Across all three tested speeds, participants were attached 37.9 ± 23.7 (T2) vs. 92.3% ± 8.4% (T4) of the time [F(1,15) = 18.73, $\eta_{p}^{2\;}\;=\;0.56$, *p* = 0.001]. Each pairwise comparison between step and LRC sounds within speed conditions was significant (*p* < 0.001). There was a moderate nonsignificant difference in BRV between conditions [21.5 ± 17.2 vs. 8.01% ± 3.31%; F(1,15) = 0.06, $\eta_{p}^{2}\;<\;0.01$, *p* = 0.81]; deeper analysis revealed a significant difference between participants (*p* = 0.03), and pairwise differences at the 90% and 100% speeds (*p* = 0.007). Finally, although there was a negligible difference in reset breaths between conditions [F(1,15) = 0.13, $\eta_{p}^{2}\;=\;0.01$, *p* = 0.72], there was a significant pairwise difference between T2 and T4 in the 110% speed condition (*p* = 0.017).

#### 3.2.2. Phase coupling

There was a large nonsignificant difference in expiration entrainment stability between conditions [F(1,15) = 4.35, $\eta_{p}^{2\;}\;=\;0.22$, *p* = 0.061], and each pairwise comparison between speed conditions was significant (*p* < 0.02). A moderate nonsignificant difference in inspiration entrainment stability [F(1,15) = 1.13, $\eta_{p}^{2\;}\;=\;0.07$, *p* = 0.31] was accompanied by significant pairwise differences at 90% and 100% speeds (*p* = 0.005). No meaningful differences were observed in entrainment timing *θ* between conditions, speed conditions, or participants.

#### 3.2.3. Within-trial T4 LRC guidance analysis

A significant, large effect of odd vs. even ratio was observed for BRV [F(1,15) = 8.01, $\eta_{p}^{2\;}\;=\;0.36$, *p* = 0.011], which was higher for odd ratio adherence (9.2 ± 1.6 vs. 5.6% ± 2.2%). No other effects upon frequency or phase coupling adherence were detected within T4 between speeds, instructed ratio, or BR deviation.

## 4. Discussion

This is one of few studies published on LRC in the field, and that examined individualized breath guidance during exercise. While the results show clear adherence to the guidance overall, there were large inter-individual differences within this sample. We attribute this varied response to small group differences in running experience, and large differences in music experience. This should be explored further in future studies.

### 4.1. Frequency coupling results in context

We found that this cohort was coupling for 26.3% ± 10.7% of the outdoor T1 run, across variable ratios (Figure 3). While this is significantly lower than the T3 outdoor run, the value of this comparison is only between step-only and LRC sound guidance, without a distinct no-sound control condition. Hence, we used the attachment analysis to retrospectively evaluate the prevalence of LRC amongst another dataset of novice female runners in a self-paced outdoor run of 45 min recorded by our laboratory (50). We found that this cohort was coupling 37.5% ± 12.8% of their runs with no instruction (51). This comparison between studies suggests that the runners in the current study were coupling less with step-only sounds than similar runners in another study with no step or breathing guidance. In contrast to the study mentioned above, and that of experienced male runners in a half marathon by Harbour et. al (52)., we observed lower LRC prevalence in the current step-only sound conditions vs. the aforementioned observational studies. We suspect that this low LRC occurrence in T1 is due to 1. the relatively low running experience of our sample and 2. the focus on step sounds possibly perturbing any natural breathing patterns.

There is limited literature with which to compare our in-field LRC instruction success. Only one similar study could be found; they also leveraged real-time SR together with haptic feedback, but reported very low adherence around 26% “success ratio” across full, intermittent, and self-selected feedback conditions (53). When calculating the same success ratio for our data, we found an average 72% ± 16%, which strongly favors our auditory approach for guiding this promising breathing strategy.

### 4.2. Phase coupling

#### 4.2.1. Relation to frequency coupling

The within-attachment analysis strongly suggests that stable frequency coupling inherently induces consistent phase coupling. While intuitive, these two components of coupling are phenomenologically distinct. It is possible to have one without the other, for example during weak frequency coupling, or, as previously reported, during alpine skiing when strong phase coupling can exist without frequency coupling (54). We propose that frequency coupling guidance that maximizes attachment is sufficient to trigger phase coupling in most runners. This could lower the barrier to creating biofeedback application using this construct, as precisely phase-synchronized sound feedback may not be needed.

#### 4.2.2. Phase lag

The mean resultant vector direction *θ* results indicate a consistently large phase lag between step (footstrike) and breath flow reversal (FR) with LRC guidance (Figure 5). It is unclear whether this reflects actual phase coupling error by the runners, or if this is due to LRC sensor detection error. If the former is true, this contradicts our hypothesis that runners would perform phase coupling with step and breath in-phase. It could be that participants were breathing correctly (in-phase) to the sound guidance but stepping antiphase to the step sounds. This was observed in at least one participant during the indoor T4 treadmill runs. Indeed, other studies have demonstrated that runners often anticipate sound cues with steps (55). Unfortunately, the attempts to measure music-step synchronization in this study were not possible due to clock drift and very small time magnitudes in question. Conversely, this phase lag could be explained by the visceral piston hypothesis. While footstrike is a key event in the mechanistic determinants of LRC, it is actually the downward momentum of the abdominal viscera, pulling on the diaphragm, which is suggested to cause step-driven flows when synchronized with FR (16). This peak downward momentum generally occurs with a large delay after footstrike (34). While the majority of studies on LRC calculate relative phase using footstrike, future studies should investigate the visceral piston timing relative to footstrike in order to improve such analysis.

This discrepancy highlights a limitation in our study–there is, to our knowledge, no currently accepted reference system or events from which to calculate LRC. Thus, it is difficult to quantify the sensor detection accuracy of the HX vs. a reference for phase coupling analysis. Nonetheless, we previously validated the FR and step detection of the HX relative to a reference spirometer and tibia-mounted accelerometer and reported event detection errors of 0.018 ± 0.086 and −0.037 ± 0.069 s, respectively (41, 43). Using the mean BR = 38 and SR = 166 observed in T1 combined with these reported event detection errors, an average phase coupling error of ±54° could be expected. This error certainly contributes noise to the phase coupling estimations compared here but cannot fully explain the systematic phase lag observed in the data.

### 4.3. Breathing variability: reset breaths

Initially, we theorized that reset breaths represent normal BRV, and would be elevated when the runner experiences respiratory distress or physical fatigue. However, during this study we observed that some individuals consciously alter their breath (i.e., breath holding) when trying to adhere to breath guidance. Thus, reset breaths might represent a psychophysiological reset or a conscious attempt to re-adhere to instructions. In post-hoc analyses, we found that the largest percentage of reset breaths occurred within 10 s of a detachment (33.9%), while many “caused” the end of an attachment (21.3%). 27% occurred away from these *a priori* margins, thus likely representing true psychophysiological resets (sighing). We attribute the lack of detectable differences in reset breaths between conditions to small sample size and large inter-individual variation; more studies are needed to understand this variable and normal values in runners.

### 4.4. Effects upon step rate

Another aspect to consider is the bidirectional relationship between step and breath rhythms previously observed by Hoffmann, Torregrosa (11). Rather, LRC primarily entrains BR to SR, but also marginally influences SR dynamics. We found no statistical differences between T1 and T3 step rate variability (3.5 ± 1.9 vs. 4.3% ± 3.6%) or any changes between run quartiles. These data appear similar to the studies mentioned above (4.3 ± 0.9 (50) and 3.5% ± 1.4% (52)), so we conclude that the step-only and LRC sound guidance did not substantially influence SR within this cohort.

### 4.5. Limitations

This study lacked randomization and a true control condition. We chose a sequential design to introduce step sounds before LRC guidance, since LRC guidance alone could be considered a dual-task problem (following step and breath cues) (35). The first visits served to familiarize runners to the synchronized step sound. Then, to precisely evaluate frequency and phase coupling, only breath guidance was added for the T3 and T4 visits. We chose a step-only sound condition instead of no sound in the first study visits in order to isolate the effects of coupled breath guidance amongst the diverse effects of synchronized step sounds. The addition of an initial no sound run may have revealed if there were any differences in LRC vs. the step sound conditions. Finally, this design was sequential instead of randomized since exposure to LRC guidance might cause learning or retention of LRC during subsequent runs; future studies could explore this topic.

The verbal familiarization during the T3 and T4 conditions had an unknown contribution to the large increase in LRC observed in those runs. While the runners only received a brief verbal familiarization to under the guidance at run onset, it likely influenced the adherence in later parts of the run. Regardless, for this study we considered instruction understanding critically important, so this aspect was not studied independently. Future studies could examine the difference between verbal-only vs. sound-only LRC instruction.

These results and statistical conclusions should be interpreted with caution since this study included a relatively small sample size (*n* = 16) and the individualization protocol led to diverse LRC ratio exposure between runners. A larger sample size and homogenous parameter selection (i.e., LRC ratios, running speeds) might have led to larger effects or more generalizable results. Previous reports suggest that runners with higher intrinsic variability decrease their variability with sound guidance, while those with lower intrinsic variability respond opposite (increase) (56). Additionally, the use of continuous sound guidance during the running protocol conflicts with current best practice in feedback learning. Recent reviews suggest that continuous feedback can create dependency and hinder learning, whereas bandwidth (feedback only when the performance is out of acceptable range) or self-determined frequency feedback are recommended to maximize learning and motivation (57).

As stated above in the section “phase coupling”, the accuracy of the HX for estimating LRC (especially phase coupling) is not entirely clear. While we have published data on its suitability for detecting the component events (FR and footstrike), there are methodological barriers to evaluating overall LRC detection vs. a gold standard. Future studies should clarify the key events from which to calculate synchronization (e.g., foot initial contact vs. peak impact) and assess such events vs. an appropriate, high sample rate reference system.

### 4.6. Application and next steps

These results show promise that a mobile guidance system can be used in the field to further the understanding and study of LRC and its potential benefits. First it was critical to report and understand deeply what, when, and how runners adhered to the sound guidance amongst various constraints. We did collect additional questionnaire, physiological, and performance data and plan to explore these in another study, for example using the within-attachment approach described above. This study design proved successful in inducing strong LRC in an acute setting and can also be used to compare the short-term effects of LRC and its implication for metabolic and perceptual responses.

Future iterations of the Breathtool app are planned to include features reflecting key learning from this study. First, we hope to provide different soundscapes and temporality options for LRC instruction. Overall, it will be designed for greater understanding and aesthetics to be used independently during longer intervention studies.

## 5. Conclusion

This study demonstrated how step-adaptive LRC sound guidance can be used to individualize breathing guidance and to strongly increase frequency and phase coupling in novice female runners. More investigation is needed to clarify the advantages and challenges of instructing this breathing strategy in the field over longer time periods, for example in an intervention study. Progressive development of this custom application is in progress to enable such studies in the wild and to maximize usability for all runners.

## Data Availability

The raw data supporting the conclusions of this article will be made available by the authors, without undue reservation.
